# Climate-mediated food safety hazards of aquaculture

**DOI:** 10.1088/2976-601x/ae854f

**Published:** 2026-07-23

**Authors:** William D Fairfield, Nilendra K Nair, Dane Klinger, Christopher D Golden

**Affiliations:** 1 Department of Nutrition, Harvard T.H. Chan School of Public Health, Boston, MA, United States of America; 2 Department of Global Health and Population, Harvard T.H. Chan School of Public Health, Boston, MA, United States of America; 3 Center for Regenerative Economies, Conservation International, Arlington, VA, United States of America; 4 Department of Environmental Health, Harvard T.H. Chan School of Public Health, Boston, MA, United States of America; 5 Indicates joint first authorship.

**Keywords:** aquaculture, climate change, food safety, pathogens, harmful algal blooms (HABs), heavy metals, planetary health

## Abstract

Aquaculture has rapidly expanded as a critical source of global food production, contributing significantly to nutrition and livelihoods. However, climate change poses substantial challenges to aquaculture, particularly in terms of food safety. This review synthesizes the literature on the impact of climate-mediated environmental changes on food safety hazards in aquaculture, focusing on biological and chemical contaminants. Rising temperatures, altered salinity, and extreme weather are amplifying the proliferation of pathogens such as *Vibrio spp*., algal biotoxins, and chemical pollutants like heavy metals and microplastics. These changes jeopardize the safety and sustainability of aquaculture products, especially in climate-vulnerable regions. Our findings highlight the urgent need for adaptive strategies to mitigate these risks, including enhanced monitoring, climate-smart aquaculture practices, and regulatory interventions. By examining the intricate relationship between climate change and food safety, this review calls for a comprehensive approach to safeguard human health and ecosystem resilience, ensuring that aquaculture can meet the demands of a growing global population.

## 1. Introduction

Aquaculture is a diverse industry encompassing the production of finfish, crustaceans, mollusks, algae, aquatic plants and other organisms (FAO 2022). Globally, per capita consumption of aquatic food has more than doubled, rising from approximately 9.0 kg in 1961 to 20.7 kg in 2022 (Naylor *et al* 2021, FAO 2024). With a majority of global wild fish stocks fully exploited or depleted due to overfishing, the increasing demand for aquatic food from an expanding, wealthier global population has contributed to a rapid increase in aquaculture production. Between 2001 and 2020, global aquatic animal production increased by 169% to reach 122.6 million tons (Mt) in live weight, comprising 87.5 Mt of aquatic animals and 35.1 Mt of algae and aquatic plants in 2020 (FAO 2022). In 2022, this figure rose to 185 Mt, increasing another 4% from 2020. For the first time in 2022, aquaculture has passed capture fisheries, accounting for 51% of the production of aquatic animals (FAO 2024). Although the rapid proliferation of aquaculture has been touted as a means to alleviate global malnutrition (Golden *et al* 2021), there exists substantial variability in both the environmental performance and nutritional value of aquatic products.

Aquatic foods, the majority of which are farmed, are an important global food resource. They contribute approximately 20% to the daily animal protein intake of an estimated 3 billion people (Golden *et al* 2021) and serve as a rich source of income and micronutrients, especially for the world’s poorest populations (Short *et al* 2021). Seafood consumption is associated with a reduced risk of cardiovascular disease due to its low saturated fat content and abundance of polyunsaturated omega-3 fatty acids (Rimm *et al* 2018). Despite many nutritional benefits, aquatic foods also pose potential risks to human health. Aquatic foods are susceptible to contamination by biological agents, including naturally occurring pathogens such as various species of bacteria and viruses, as well as to the accumulation of heavy metals and agrochemical residues from pollution and anthropogenic activity (Buzina *et al* 1989, Anater *et al* 2016, Stentiford *et al* 2022).

Environmental factors such as precipitation, water temperature, salinity, and pH serve as drivers of water and foodborne contamination and illnesses. While these factors are not the direct source of contamination, they permit contamination to occur in greater quantities and in new regions, furthering food safety risk. Anthropogenic climate change impacts these factors, thereby posing a threat to both the growth of aquaculture and the safety of its products (Schiedek *et al* 2007). This threat to the sustainability, growth, and safety of aquaculture and seafood is particularly concerning for countries located in climate-vulnerable regions, including top-producing countries such as China, India, Indonesia, and Vietnam, which account for 36%, 8%, 7%, 5% of global aquaculture production, respectively (FAO 2024). There are many primary studies on the implications of climate change on the food safety risks of aquatic products. However, there is a lack of synthesis of this information to show larger trends that may affect the future of aquaculture.

In this review, we present a synthesis of the published research on how climate change and industrialization might alter the risks associated with the seafood safety of aquaculture products. Our findings underscore the intricate interplay among contaminants, climate change dynamics, and their repercussions on seafood safety, emphasizing the urgent need for mitigation strategies and vigilance in monitoring to safeguard both human health and aquatic ecosystems.

## 2. Methods

### 2.1. Literature search and screening

The methodology for this review included five key components: (1) identifying the research question, (2) parsimonious selection of keywords and descriptors to guide the literature review, (3) identifying relevant studies, (4) quality control and screening, and (5) collating and summarizing the results.

The initial retrospective search was implemented in August 2020 in the Web of Science (WoS) electronic database using key descriptors (supplementary information—S1). The search was structured around key ‘Title’ and ‘Descriptors’ to identify the corpus of relevant scientific literature. The WoS also included the world-renowned CAB Abstracts database search for relevant abstracts that had been tagged with the pre-selected descriptor keywords (supplementary information—S1). We also searched MEDLINE/PubMed and SciVerse Scopus (Elsevier) to identify any gray literature and hand-searched the references of pertinent primary studies for relevant publications.

The search criteria were constrained to primary articles published in English between 2000 and 2020, excluding duplicates, review articles, publications primarily about aquaculture feed contamination, veterinary articles regarding the health and disease of aquatic animals, articles providing experimental technological methods, articles published in dubious journals, and articles that reported on aquatic food products that are exclusively wild-caught. While review articles were excluded, we scanned their reference lists for relevant primary articles that met our inclusion criteria and may have been missed by our initial search. Follow-up searches were conducted in the WoS database in August 2021 and August 2025 to identify any additional publications after the initial search. A search of MEDLINE/PubMed and SciVerse Scopus was also conducted at this time, and the first 100 publications were screened for additional gray literature such as government, non-profit, and industry publications.

**Figure 1.**
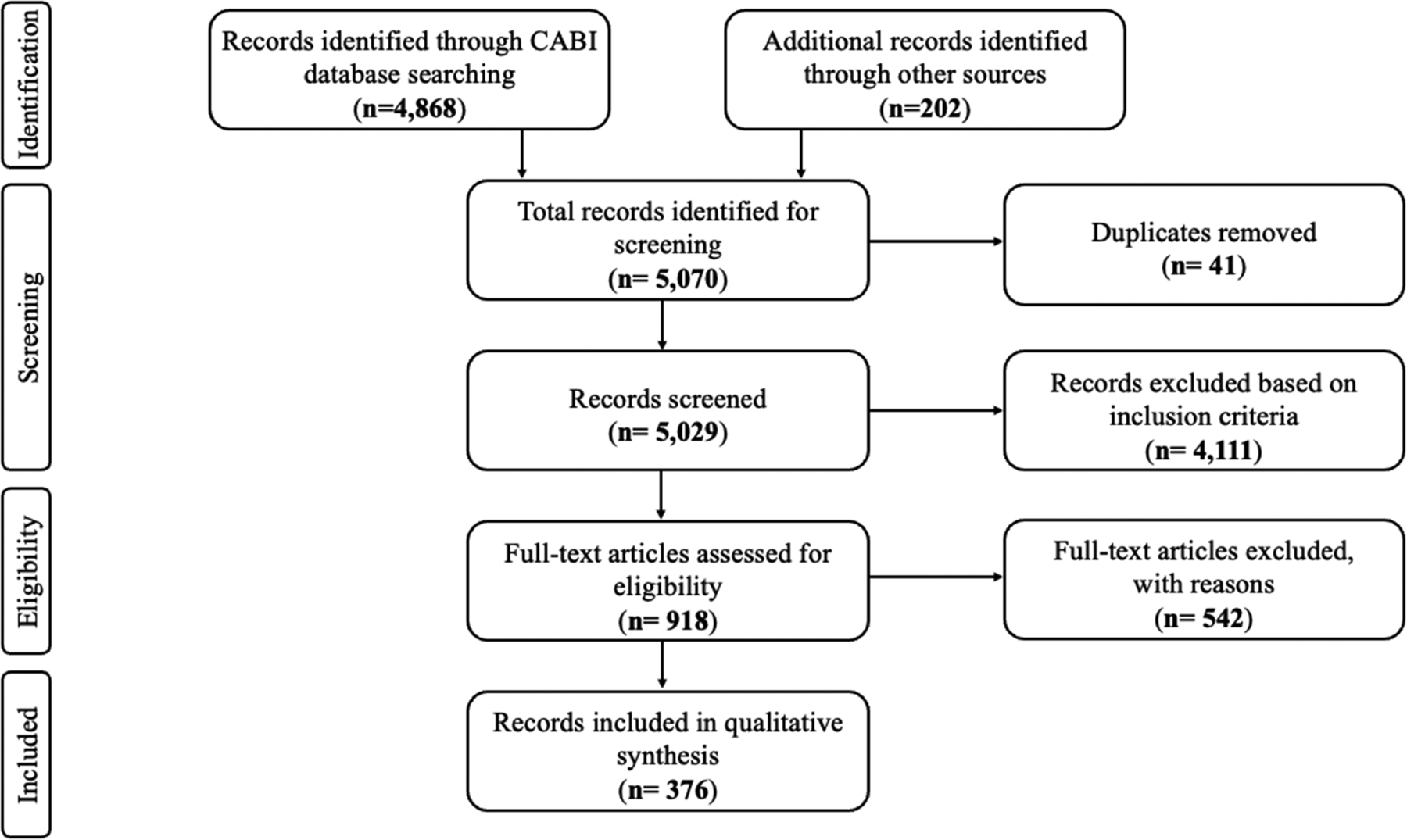
Systematic literature review screening process. Identification and filtering of included records.

A total of 5070 publications were identified: 4868 through the WoS search in the CABI database, with an additional 202 identified through a hand search of gray literature (figure 1). A two-stage screening process was employed to assess the relevance of the identified publications. Initially, duplicates were removed, and then only the titles and abstracts were screened based on the minimum inclusion and exclusion criteria to prevent the waste of resources in procuring irrelevant publications. Following the initial screening, 918 full-text articles were then assessed for eligibility, and a grand total of 376 studies were included in the qualitative synthesis in this review.

### 2.2. Data standardization, aquaculture-relevant species screening, and descriptive analysis

Species and taxon names reported in the included publications were extracted and standardized at the most specific taxonomic level possible using a multi-step cleaning and validation process. Reported names were normalized for formatting, abbreviated genus names were expanded where sufficient context existed, and apparent typographical errors, synonyms, or outdated scientific names were corrected using accepted nomenclature. When only a scientific name was reported, the corresponding common name was added. When only a broad common name was reported and species-level identification was unavailable, the entry was retained at the reported level, such as ‘Common Name (no species specified),’ rather than assigned to a presumed species. Standardized names were cross-referenced using taxonomic resources including FishBase, SeaLifeBase, and the World Register of Marine Species. Ambiguous, genus-only, or higher-level entries were retained as reported rather than over-resolved.

To support an aquaculture-focused species summary, standardized taxa were screened for aquaculture relevance. Species or commodity groups known to be primarily wild-caught and not commonly produced in aquaculture were excluded from the aquaculture-focused species and commodity-group analyses. For commodity groups that included both farmed and wild-caught species, including taxa initially retained in broader residual categories, we conducted an additional species-level review to identify taxa judged to be primarily wild-caught. These taxa were also excluded from the aquaculture-focused analysis. See supplementary information (S1), for the search terms used to filter out these records.

After the exclusion of primarily wild-caught taxa, in remaining records that were mixed or uncertain with respect to production method, the source publication was reviewed to determine whether the sampled product was identified as farmed, wild-caught, mixed, or not specified. Taxa known to occur in both farmed and wild-caught production systems were retained when the production method was identified as farmed, mixed, or not specified, but excluded when the source study identified them as wild-caught. For taxa whose aquaculture relevance remained uncertain, records were retained only when the source study supported aquaculture relevance, such as identifying the sample as farmed, cultured, or collected from aquaculture ponds, farms, cages, hatcheries, or mariculture sites. Uncertain taxa were excluded when production method was not specified and aquaculture relevance could not be confidently determined.

Remaining standardized taxa were retained for the aquaculture-focused analysis and were grouped into common aquatic food commodity categories for descriptive reporting. Commodity groups were defined pragmatically based on common seafood market names, recognizable food-system categories, taxonomic proximity where useful, and recurring high-frequency groups in the dataset. Terms used for allocation into commodity groups and for wild-caught exclusion are provided in supplementary information (S1).

For analysis, duplicate species entries within the same study were removed so that each study-species combination contributed only once. Descriptive summaries were generated for standardized species, commodity groups, contaminant categories, climate-related hazards, bacterial genera, and geographic region. Additional cross-tabulated summaries were produced to examine relationships among these categories, including interactions between contaminants, climate-related hazards, geography, and commodity groups. Analyses were conducted in R statistical software (R Core Team 2025), and a GitHub link to the complete set of summary tables and analysis script is provided in the supplementary information.

## 3. Results

**Figure 2.**
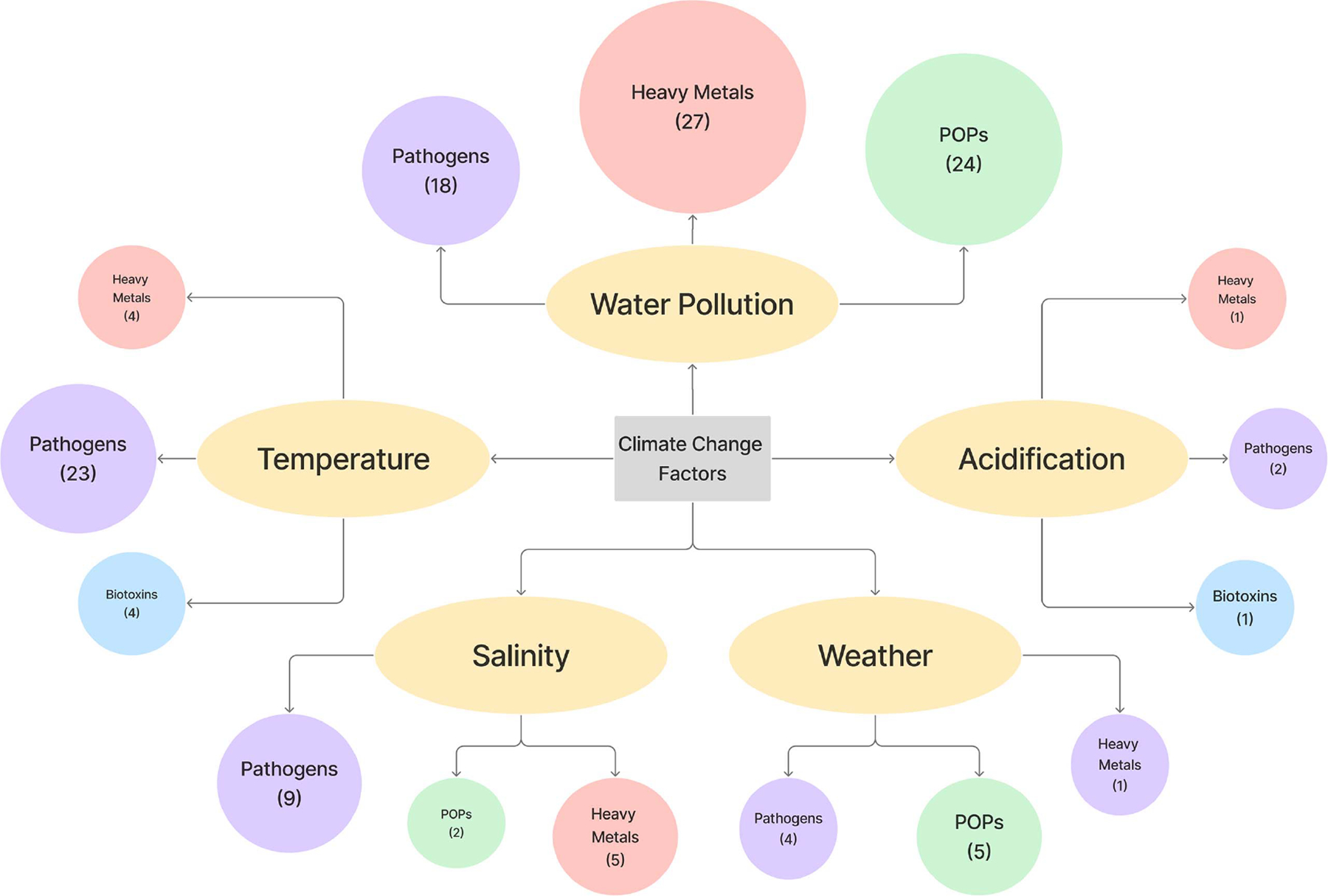
Climate change factors influence specific categories of food safety risk. This figure displays the susceptibility of environmental determinants of seafood safety to climate change and the categories of food safety risk we focused on in this review. The size of the circle corresponds to the frequency of interactions between the climate change factor and food safety risk. Additionally, the number of studies citing the interaction is included below the food safety risk. Pathogens refer to bacterial, viral, and parasitic agents.

Through our literature review and screening process, we identified 376 studies to include in this synthesis (see Methods). These studies, ranging from 2020–2025, incorporate analyses of samples from 64 countries and 8 bodies of water. The countries most frequently represented are China (75 studies), India (37), Spain (21), Italy (20) and South Korea (13). A full geographic summary can be found in supplementary data file (S2). Our review of the eligible and selected literature revealed two main categories of food risk related to both seafood safety and climate change: biological and chemical contaminants. The major biological contaminants include aquatic biotoxins (17 studies), and pathogens (137) which include bacteria, viruses, and parasites. The main chemical contaminants, originating from both human activities and natural sources, include heavy metals (104), persistent organic pollutants (POPs) (90), microplastics (30), and antimicrobials (9). In this synthesis, we have compiled the current understanding of the origins of these contaminants, their implications for human health, and their potential interactions with environmental factors amidst a changing climate (figure 2).

### 3.1. Commodity group coverage

After cleaning and analyzing, the dataset included 1395 study-species occurrences representing 417 unique standardized taxa. Species-level frequencies were first calculated using standardized taxon names, with each species counted only once per study. Standardized species and frequencies can be found in the supplementary data (S2). The most frequently represented commodity groups were Clam/Cockle, Seabass/Sea Bream, Mussel, Cyprinids (Carp/Minnows), and Oyster appearing 242, 157, 125, 103, and 99 times across the review (table 1). Additional species-specific information can be found in the supplementary data (S2).

### 3.2. Biological contaminants and seafood safety in a changing climate

Biological contaminants in seafood are naturally occurring pathogenic microorganisms capable of causing illness when consumed in contaminated seafood products. Contamination by biological agents can be attributed to biotoxins produced by harmful algal blooms (HABs), along with the infection and contamination of seafood by naturally occurring microbial, viral, and parasitic agents. The sections below examine the risks associated with consuming aquaculture products contaminated by these biological elements and explore the interactions between these pathogens and climate change factors.

#### 3.2.1. Biotoxins

Out of 17 studies in our review that reported biotoxin contamination of aquatic food products, 4 reported links to human health risk. Biotoxins were co-reported along with multiple climate change factors, namely water pollution (5 studies), temperature (4), salinity (2), and acidification (1).

Aquatic biotoxins pose a significant threat to aquaculture and seafood safety. HABs involve the proliferation of toxic phytoplankton (e.g. diatoms and dinoflagellates) that produce phycotoxins (e.g. ciguatoxins) and cyanobacteria/blue-green algae which produce cyanotoxins (e.g. saxitoxins or lyngbyatoxin-a) in both freshwater and marine environments (Tamele *et al* 2019). These biotoxins, originating from microalgae, accumulate in shellfish and filter-feeding fish, subsequently transferring to organisms higher in the food chain (Mulvenna *et al* 2012). These HAB toxins are responsible for illnesses such as Ciguatera fish poisoning (caused by ciguatoxin), which is the most common seafood-related illness associated with HABs, although it remains rare in farmed fish (Grattan *et al* 2016). Other associated illnesses include various types of shellfish poisoning (e.g. paralytic, amnesic, azaspiracid, neurotoxic, and diarrhetic). Common acute symptoms resulting from the ingestion of seafood contaminated by biotoxins include nausea, vomiting, diarrhea, numbing or tingling of the mouth, face, and neck, short-term memory deficits, and gastroenteritis in humans (Glibert *et al* 2005).

HABs are a naturally occurring phenomenon. Nevertheless, the increased frequency, extended duration, and expanded geographical range of these blooms have largely been attributed to increased anthropogenic activities. Factors contributing to these increases include anthropogenic climate change and runoff from urban and agricultural areas, which contribute to nutrient enrichment and eutrophication (Glibert *et al* 2005) (table 2). Climate change has led to rises in temperature, increased nutrient loads, and a re-distribution of nutrient sources and sinks that lead to HAB proliferation (Glibert *et al* 2005) (table 2). In recent years, increasing amounts of evidence point toward how climate change may influence toxin production. Salinity (Osburn *et al* 2023, Shi *et al* 2024) and temperature (Farrell *et al* 2015, Liu *et al* 2025) both affect the metabolism of harmful algae.

#### 3.2.2. Bacteria

Pathogen contamination of aquatic food products, including bacteria, viruses, and parasites, was reported 137 studies in our review. 33 studies reported links to human health risk while co-reports along with climate change factors include temperature (23 studies), water pollution (18), salinity (9), extreme weather (4), and acidification (2).

Contamination of aquaculture by microbial pathogens such as bacteria, viruses, and parasites can pose a pathogenic risk to humans when consuming aquatic food products. Bacterial pathogens can be categorized either as those that are naturally occurring in the aquaculture environment (e.g. members of the *Vibrio* spp) (Bonnin-Jusserand *et al* 2019) or as those introduced into the environment, usually through fecal contamination (e.g. *E. coli, Salmonella* spp.) (Amagliani *et al* 2012). 122 out of 376 studies included in this review reported bacterial contamination of aquatic food products. Of these, 57 studies reported members of the *Vibrio* genus as a specific risk factor. We also found notable accounts of *Escherichia, Salmonella, Staphylococcus,* and *Listeria* genera which are reported in 21, 20, 18, and 14 studies respectively. Summary of all bacterial genera can be found in the supplementary data (S2).

Outbreaks and infections caused by members of the *Vibrio* genus (*V. cholerae, V. vulnificus, and V. parahaemolyticus*) are among the most common and extensively studied foodborne infections, with symptoms ranging from gastroenteritis to more serious neurological and renal syndromes (Strom and Paranjpye 2000). Foodborne infection from *Vibrio* spp. rose from 0.7 cases per 100 000 people in 2016–2018 to 1.1 cases per 100 000 in 2023 (Shah *et al* 2024). While it is difficult to determine how many of these cases result from eating farmed organisms, this increasing trend and the rising contribution of aquaculture to global seafood consumption necessitates that vigilance is maintained toward *Vibrio* spp. outbreaks in aquaculture. Additionally, aquaculture is characterized as a hotbed for gene transfer (Watts *et al* 2017). With increasing incidence of antibiotic resistant genes (ARGs) in *Vibrio* spp., and their transfer to seafood (Dong *et al* 2025), it is important to understand *Vibrio* spp. and associated ARG dynamics in aquaculture contexts.

The ecology of *Vibrio* spp. is predominantly associated with coastal estuarine and marine environments, where proliferation dynamics are mainly influenced by seawater temperature and salinity levels (Randa *et al* 2004). Members of this pathogenic family generally thrive within temperatures ranging from 16 °C–33 °C and a salinity level of 5–20 ppt (Wetz *et al* 2014). The pathogen’s optimal habitat is defined by the salinity levels, while the temperature regulates its growth and abundance within these optimal habitats. Reports suggest that climate change has significantly increased the occurrence of *Vibrio* worldwide, with the pathogen being detected in unusual or previously rare areas (Baker-Austin *et al* 2010). Rise in temperature, along with a decrease in coastal water salinity, provides optimal environmental conditions for the growth and proliferation of *Vibrio* (table 2). Further climate changes are likely to lead to the proliferation of *Vibrio* beyond its usual geographical range (Sampaio *et al* 2022).

#### 3.2.3. Viruses and parasites

Like bacterial agents, viruses and parasites are found in both farmed fish and seafood products, presenting potential health risks to consumers and leading to various foodborne illnesses and infections. Viral diseases pose a significant concern in aquaculture due to their capacity to cause mass mortalities and result in substantial economic losses. Among the most well-known viruses affecting farmed fish is the infectious salmon anemia virus (Nylund *et al* 2019), viral nervous necrosis virus in marine fish, and the infectious pancreatic necrosis virus found in both marine and freshwater fish species (Wallace *et al* 2008, Yang *et al* 2022). There are other viruses that can infect humans, leading to morbidity and mortality. For instance, consuming raw or undercooked shellfish can lead to norovirus and Hepatitis A (Jothikumar *et al* 2005). Infections caused by nematodes, trematodes, and cestodes are also caused by the consumption of raw or undercooked seafood. Anisakiasis, a parasitic infection caused by nematodes or roundworms of the *Anisakis* genus, arises from the consumption of infected aquatic food products and can result in invasion of the stomach wall and intestines.

Climate change is significantly impacting aquaculture and seafood products by influencing the distribution and prevalence of viruses and parasites. Rising temperatures and changing environmental conditions are altering disease patterns, enhancing viral replication, and disrupting the life cycles of parasites. Elevated water temperatures due to climate change can lead to the emergence and spread of new and more virulent pathogens in aquaculture and seafood products (Lafferty *et al* 2015). Additionally, shifting ocean currents influence the prevalence and distribution of viruses and parasites in aquaculture settings (table 2). Warmer waters can accelerate the replication rates of certain viruses and parasites, thereby escalating disease transmission and fostering outbreaks (Lafferty *et al* 2015) (table 2). Moreover, certain pathogens may expand their range to new areas, affecting aquaculture facilities previously not exposed to such risks.

### 3.3. Chemical contaminants and seafood safety and the impact of climate change

A second major threat to seafood safety is posed by chemical contaminants present in the aquaculture environment. These contaminants consist of both organic and inorganic pollutants predominantly originating from anthropogenic activities over the past century. These include POPs, toxic metals, synthetic organic chemicals, and industrial pollutants. Here, we outline the evidence concerning the risks to seafood safety posed by chemical contaminants as well as the influence of climate change on the exposure risk to these pollutants in relation to aquaculture production and human health.

#### 3.3.1. Persistent Organic Pollutants (POPs)

POP contamination of aquatic food products was reported 90 studies in our review. 31 of these reported links to human health risk and co-reports along with climate change factors include water pollution (24 studies), extreme weather (5), temperature (3), salinity (2), and acidification (1).

POPs constitute a broad class of halogenated, stable organic compounds that are resistant to environmental degradation. These compounds exhibit low decay rates and demonstrate resistance to photolytic and biological breakdown in aquatic environments, leading to increased concentrations of POPs and a higher potential for bioaccumulation (Latimer and Zheng 2003). Although some POPs can be attributed to natural sources, many of these contaminants arise from increased anthropogenic activities, entering aquatic environments through atmospheric deposition, discharge of industrial chemical waste and effluents into water sources, and pesticide runoff (Latimer and Zheng 2003, Guo *et al* 2019). POPs can lead to endocrine disruption, affecting normal hormone homeostasis and leading to cancer and other adverse health effects in both mammals and humans (Guo *et al* 2019).

Climate change factors such as increased temperatures and intensified storms and winds impact an important geochemical process called global distillation (Sadler and Connell 2012). This phenomenon, also known as the grasshopper effect, causes the transfer of POPs from warmer regions to cooler parts of the earth (Sadler and Connell 2012). In fact, despite the limited use of these compounds in polar regions such as the Arctic and the Antarctic, these areas bear a higher burden of POP exposure (de Wit *et al* 2022). Additionally, rising temperatures, ultraviolet (UV) radiation, and increased precipitation contribute to the enhanced volatilization of POPs from primary emission sources and the re-volatilization of these substances deposited in soil and sea-ice (de Wit *et al* 2022), increasing both the bioavailability and toxicity of these compounds to aquatic organisms (Noyes *et al* 2009) (table 2). The rise in salinity has the potential to affect either the contaminants themselves or the physiology of aquatic organisms and their ability to regulate toxicity (Noyes *et al* 2009).

#### 3.3.2. Antimicrobial agents

Antimicrobial contamination of aquatic food products, which includes pollution from drugs used in the aquaculture environment, was reported 9 studies in our review. Only one of these reported links to human health risk while the co-reports along with climate change factors include water pollution (2 studies) and temperature (1).

The increase in bacterial pathogens due to the intensification of aquaculture practices has led to a surge in antimicrobial usage (Ibrahim *et al* 2020). Antimicrobial agents are usually introduced prophylactically to prevent infections, therapeutically to manage infectious outbreaks, or metaphylactically to promote growth of cultured animals (Schar *et al* 2020). In a recent evaluation of global trends in antimicrobial usage for humans, terrestrial food-producing animals, and aquaculture, Schar *et al* estimated a global antimicrobial consumption in the aquaculture industry of approximately 10 259 tons in 2017 with a projected increase of approximately 33% by 2030, reaching about 13 600 tons (Schar *et al* 2020). Although this future projection of antimicrobial use for aquatic food-producing animals would account for only 5.7% of the overall global consumption of medically important antimicrobials, its application in aquaculture carries the highest intensity per kilogram of biomass, approximately 164.6 mg kg^−1^ (Schar *et al* 2020). The diverse applications of antimicrobial agents have led to an increase in pharmaceutical contaminants in the aquatic environment, potentially contributing to the surge in antimicrobial resistance among bacterial strains in the water column, sediment, and cultured species (Cabello 2006, Cabello *et al* 2016, Okocha *et al* 2018).

Higher temperatures are known to induce stress and compromise the immune system of many aquatic species, rendering them susceptible to infection and leading to increased rates of mortality (Ashrafi *et al* 2018). As temperatures rise and other climatic and environmental factors shift, emerging infectious diseases are forecasted to increase due to increased pathogen growth and virulence (Cabello *et al* 2016, Ashrafi *et al* 2018) (table 2). In 2017, countries in the Asia-Pacific region, mainly China, India, Indonesia, and Vietnam, were the highest consumers of antimicrobials, concurrently holding the largest share of aquatic products (excluding mollusks) (Ibrahim *et al* 2020).

#### 3.3.3. Heavy metals

Heavy metal contamination was represented substantially in our review with 104 studies reporting it. 36 of these reported links to human health risk, and co-reports along with climate change factors include water pollution (27 studies), salinity (5), temperature (4), extreme weather (1), and acidification (1).

Metals naturally occur in the environment due to processes such as weathering of rocks and volcanic eruptions (Bradl 2005). These metals are categorized as essential, non-essential, or toxic in relation to human health. Essential metals such as copper, iron, selenium, and zinc play crucial roles in metabolism, whereas toxic heavy metals such as lead, mercury, and arsenic can pose harmful effects on human health beyond certain thresholds (Mertz 2009). The ecotoxicology of heavy metals is a significant concern due to their non-biodegradable nature, ability to bioaccumulate, and inherent toxicity at high concentrations (Bradl 2005).

The presence and concentration of heavy metals in aquatic ecosystems, and consequent exposure to aquaculture species, are exacerbated by anthropogenic activities such as industrial pollution, agricultural intensification, and waste disposal (Bradl 2005). Aquaculture species are typically exposed to toxic heavy metals through contaminated feeds, metal reservoirs in aquatic sediment, and aquatic ecosystems. These metals eventually assimilate into the organism and, when the concentration of heavy metals surpasses the tolerable thresholds, they pose a serious risk to both aquatic and human health.

Climate-related variables have been reported to affect the transport, deposition, and transfer of metal contaminants in the marine environment (Iordache *et al* 2022). The bioavailability of metals is greatly affected by environmental levels, where reduced or lower salinity levels have been shown to increase metal uptake by crustaceans and mollusks. Higher temperatures were noted to increase metabolism and subsequent uptake of metal contaminants (Wijngaard *et al* 2017) (table 2). Broad investigations into the interactions between various abiotic environment factors and metals have highlighted the increased toxicity with rising temperatures (Heugens *et al* 2001). Additionally, changes in precipitation patterns, resulting in intense and frequent rainfall, are predicted to increase the runoff of heavy metals from soil, agricultural land, or landfills into water ecosystems. In parallel, adverse weather events, such as prolonged droughts, have also been observed to influence the bioaccumulation of metals in aquatic organisms (Wijngaard *et al* 2017), leading to upwards of 20% increases in the muscle tissue concentration of methylmercury under drought conditions compared to the previous year under normal conditions (Azevedo *et al* 2018) (table 2).

There is a paucity of literature that has adequately determined the bioavailability and potential toxicity resulting from consumption of metal-contaminated aquatic foods. Investigators have indicated that, on average, moderate to recommended intakes of seafood may not pose a concern; however, the potential adverse health risks for frequent consumers cannot be discounted (Djedjibegovic *et al* 2020). Documented cases of metal poisoning resulting from overconsumption of seafood products, such as methylmercury poisoning due to excessive consumption of tuna (Ahn *et al* 2018), have been reported. Shellfish have been well studied for their ability to bioaccumulate metals such as arsenic and cadmium (Olmedo *et al* 2013). Symptoms of acute metal ingestion usually include diarrhea, fatigue, nausea, vomiting, abdominal pain, confusion, and hallucinations, with neurotoxicity and carcinogenesis as extreme possibilities (Barchiesi *et al* 2020).

#### 3.3.4. Microplastics

Microplastic contamination of aquatic food products was reported in 30 studies. Five of these reported links to human health risk, and co-reports along with climate change factors only included water pollution which was reported in 12 studies.

Microplastics have achieved an unprecedented ubiquity in our biophysical environment since their upscaled production in the early 1960s (Thompson *et al* 2004). The sources of microplastics are distinguished as being either primary or secondary in nature, depending on the scale at which they were manufactured. Primary microplastics usually originate from industrial cleaning products, the production of which has been banned in the US, EU, and Canada (Koehler *et al* 2015). In contrast, secondary microplastics result from the fragmentation and degradation of plastic litter by abiotic climate factors such as sunlight (UV), temperature, pH, and weathering by wave action (Koehler *et al* 2015). The degradation of whole plastics into micro- and nano-plastics releases endogenous chemical additives that were included as part of the plastics’ manufacturing process. The hydrophobic surface of microplastics is highly amenable to adsorbing and concentrating organic contaminants (especially POPs), as well as heavy metals (Rochman *et al* 2014), making them vectors for these pollutants, which are known to have adverse health effects (Vanden Bilcke 2002).

For both marine and freshwater aquaculture systems, microplastics are ubiquitous in almost every stage of the seafood farming process (Chen *et al* 2018). The development of mariculture and the use of mostly plastic fishing and farming gear increase the availability and consequent accumulation of offshore microplastics that are readily consumed by biota. These microplastics often transfer and accumulate through various trophic levels (through the food chain), across taxa (Setälä *et al* 2014). Additionally, microplastics are found in all levels of the water column to include surface water, subsurface water, and sediments (Jolaosho *et al* 2025). The presence of microplastics in nearly all areas of aquatic organisms’ habitat further increase its exposure beyond that encountered from the use of plastic fishing and farming gear. In lab-based studies mimicking coastal ecosystems, investigators demonstrated that microplastics were ingested by all the organisms they tested, with shellfish having the highest concentrations (Setälä *et al* 2014, 2016).

The relationship between climate change and microplastics is both dynamic and reciprocal. While climatic factors such as increased temperature, pH, weathering, and UV radiation intensity lead to further breakdown of microplastics, studies have also demonstrated that microplastics are exacerbating climate change by emitting greenhouse gases when exposed to solar radiation (Ford *et al* 2022) (table 2). Additionally, microplastics are affecting the marine carbon cycle and oceanic greenhouse gas sequestration by affecting the ability of microalgae and zooplankton to photosynthesize and capture CO_2_ at the ocean’s surface (Shen *et al* 2020).

While plastics are considered to be essentially inert substances, posing little to no spontaneous chemical reactivity, upon ingestion in appreciable amounts, they affect important biological pathways that can cause system-wide issues, ranging from chronic inflammation and oxidative stress to genotoxicity, apoptosis, and carcinogenesis (Vethaak and Legler 2021). This emphasizes the potential risk of chronic exposure and consistent consumption of seafood could have on human health.

## 4. Climate change and aquaculture production

**Figure 3.**
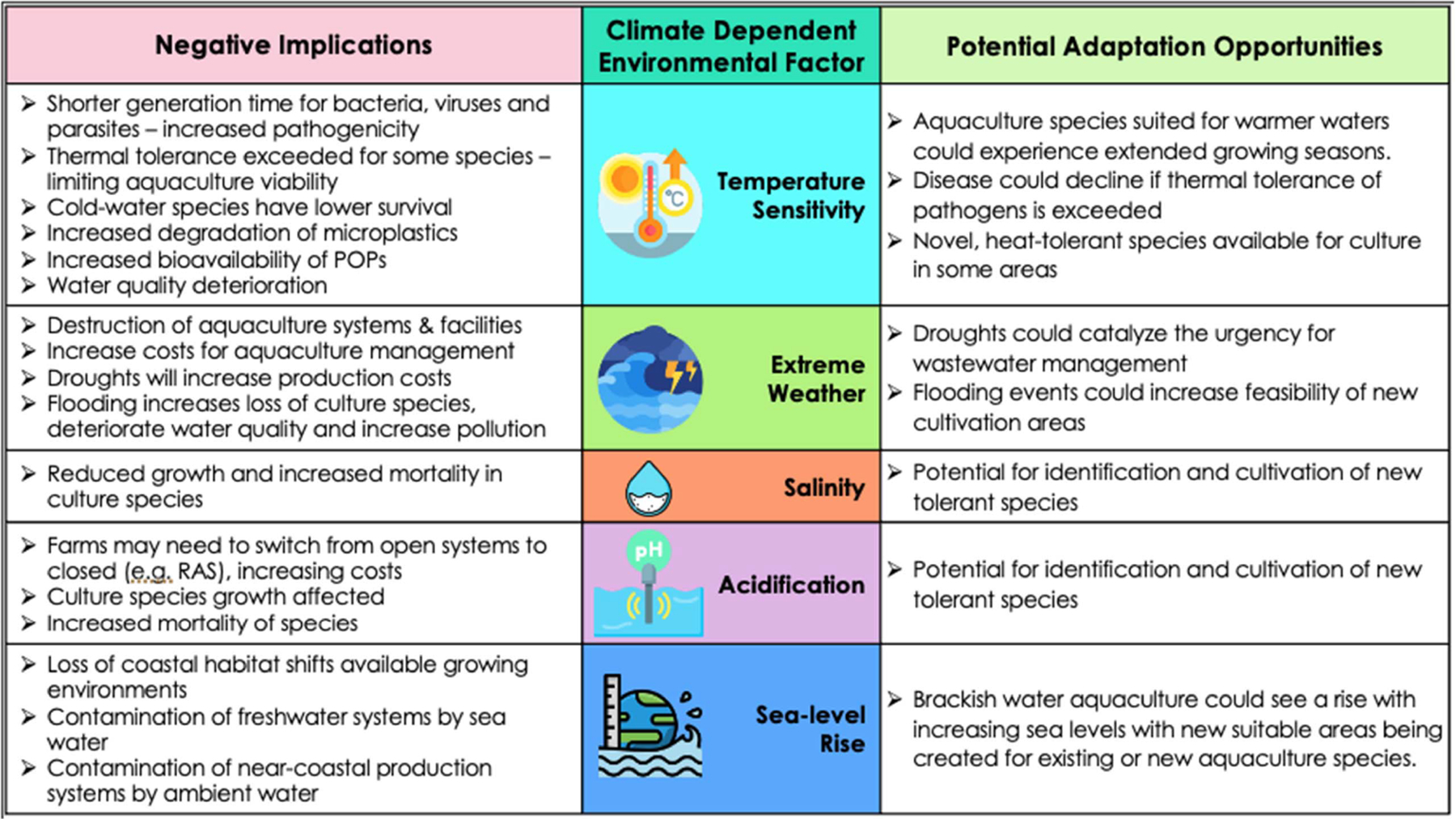
Positive and negative implications of climate-dependent environmental factors on aquaculture.

Climate change has become a pressing global issue with far-reaching consequences in the aquaculture sector (figure 3). Long-term alterations in temperature, precipitation patterns, and extreme weather events can have a profound impact on aquatic environments, affecting water quality, habitat suitability, and the overall productivity of aquaculture systems. Rising temperatures can lead to changes in water chemistry, including increased acidity and decreased oxygen levels, adversely affecting the health and growth of aquatic organisms (Mugwanya *et al* 2022). In addition, alterations in precipitation patterns can result in changes in water availability and salinity levels, further impacting the viability of aquaculture operations. Extreme weather events, such as storms and hurricanes, can cause physical damage to aquaculture infrastructure, disrupt production cycles, and lead to the loss of valuable stock (Zolnikov 2019).

Alterations in temperature, salinity, acidification, and nutrient availability in aquatic environments also have the potential to affect the quality and safety of seafood. Variations in salinity levels resulting from climate change can influence the growth and survival of HABs, which produce toxins that can accumulate in seafood. Increased ocean acidification (OA) from high atmospheric CO_2_ also has widespread negative implications for aquatic organisms (Kroeker *et al* 2010). Bivalves, in particular, experience extreme impacts from OA on their survival, development, feeding rates, and calcification (Hu *et al* 2026). Furthermore, changes in nutrient availability may affect the composition of aquatic food webs, potentially leading to the bioaccumulation of contaminants in seafood, including in fishmeal and fish oil used in aquafeeds. These climate-induced changes pose significant challenges for aquaculture practices, jeopardizing the sustainability and resilience of the industry.

While climate change poses significant challenges to various sectors, it may bring about some positive impacts on aquaculture, offering opportunities for growth. Some investigations have spurred surprising results on potentially beneficial implications of climate change on aquaculture production (figure 3). One of these positive impacts is that increased water temperature can lead to enhanced growth rates in aquatic organisms. Warmer water temperatures can accelerate the metabolic rate of fish, resulting in faster growth and development as production is currently occurring in sub-optimal temperatures; however, production will slow after optimal temperatures are exceeded with climate change (Klinger *et al* 2017). This is particularly beneficial for aquaculture, as it allows for increased production and higher yields. Additionally, this study highlights that increased water temperature can also lead to improved feed conversion efficiency, as fish are able to utilize their food more effectively. This can contribute to reducing the overall environmental impact of aquaculture operations. Therefore, the positive impact of increased water temperature on growth rates in aquaculture holds great potential for meeting the increasing global demand for seafood in a sustainable and efficient manner (Besson *et al* 2016). Additionally, the expanded distribution of species and enhanced growth and productivity offer opportunities for the aquaculture industry to adapt and thrive in a changing environment. However, to harness these benefits sustainably, aquaculture stakeholders must prioritize the adoption of climate-resilient and environmentally friendly practices. By embracing these strategies, aquaculture can continue to contribute significantly to global food security and economic prosperity in the face of ongoing climate challenges.

However, it is crucial to acknowledge that these positive impacts should not overshadow the urgent need to address the more significant overall negative effects of climate change on the sector. Marine heatwaves and increasing temperatures have devastating effects on organism survival and ecosystem stability. Climate change also alters salinity and increases OA, both of which stress aquatic species and reduce growth and survival rates (Marium *et al* 2023). It also intensifies extreme weather events and promotes disease outbreaks and HABs that damage farms and stock. As a result, productivity, food security, and the livelihoods of communities that depend on aquaculture are increasingly at risk (Islam *et al* 2022).

## 5. Discussion

While the positive impacts of climate change on aquaculture present opportunities for growth and increasingly efficient productivity, it is crucial to acknowledge and address potential challenges that may arise with climate change. These challenges will likely lead to adaptations in techniques for culturing. And, as aquaculture practices evolve in response to climate change, existing food safety regulations will need updating. Enhanced monitoring ensures that new practices are compliant with safety standards and that any emerging risks are detected and managed effectively. Producers must adopt climate-smart aquaculture practices to ensure long-term benefits while mitigating negative consequences on the environment and consumers.

A formal risk assessment for each food safety issue under climate change would require two key components—1) detailed knowledge of the baseline risk and 2) science-based estimates of how the likelihood and severity of the risk might change with expected climate change and anthropogenic development (Karunasagar 2008). Unfortunately, a lack of robust data in the aquaculture sector has inhibited a detailed characterization of baseline risk for most food safety issues in aquaculture species, production systems, and geographies (Sapkota *et al* 2008). Despite a dearth of information on these issues, generic risk mitigation and adaptation strategies are available at the farm level, sector level, and regulatory level.

### 5.1. Farm level interventions

Farms can mitigate food safety risks by transitioning between production methods and species. By switching from open to closed production systems (e.g. from cages in open water systems to recirculating tanks on land), farms can reduce exposure to changing ambient environments and increased levels of pathogens and contaminants (Lawson 1995). Differences between the two main closed production systems, recirculating aquaculture systems (RAS) and flow-through systems (FTS), should also be considered. Compared with FTS, RAS has been shown to increase heavy metal accumulation in trout and associated consumption health risk (Barszcz and Sidoruk 2024). In addition to production method, farms can switch between species, including between fed and unfed species. Despite these options, structural changes to farms or changing species often require substantial investment and may be cost prohibitive (Leung *et al* 2007, Engle 2010).

Setting up effective HAB monitoring and early warning systems involves a multi-tiered approach to detect and respond to algal blooms promptly. First, deploying a network of monitoring stations equipped with sensors that measure key indicators such as chlorophyll-a levels, nutrient concentrations, and water temperature helps in real-time detection of conditions conducive to HAB formation (Seltenrich 2014, Moron-Lopez *et al* 2021). Complementing sensor data with regular sampling and analysis of water samples for the presence of specific algal species and toxins provides additional confirmation of HAB events. Additionally, integrating satellite remote sensing technology can enhance spatial coverage, offering broader and more frequent monitoring of algal blooms across larger areas (Zahir *et al* 2024). By establishing a centralized data management system and combining real-time monitoring, advanced remote sensing, and effective communication strategies, these systems can provide timely warnings and enable prompt response actions to mitigate the impact of HABs on aquaculture and public health.

Contaminant type, exposure pathway, and impact vary substantially by region, production environment, and aquatic food species. Farms may face different risks depending on local industrial or agricultural activity, wastewater management, contaminant patterns, and available monitoring systems. Accordingly, adaptive responses such as changing production sites, modifying production methods, shifting harvest timing, or transitioning to different species should be guided by regionally relevant contaminant data where available. An adaptation strategy that reduces exposure in one setting may not be appropriate or effective in another. Additionally, there are key differences in contaminant risk for aquaculture and wild-caught operations. This includes risks associated with heavy metals (Casanova-Martínez *et al* 2025), POPs (Foran *et al* 2005), and pathogens (Sharma *et al* 2021). Therefore, it is important for farmers to have a critical lens when reviewing literature to inform their decision making.

### 5.2. Sector level interventions

At the sectoral level, producers and supply chain companies can mitigate food safety risks by investing in and developing monitoring and management tools and protocols that benefit a majority of producers. This can include trade associations, industry groups, and pre-competitive platforms that coordinate between producers and with supply chain companies to address common food safety risks (Blasiak *et al* 2021). Strategies can include development of monitoring tools for rapid detection of pathogens and contaminants (Bavisetty *et al* 2018), and development of management platforms and protocols for effectively addressing food safety risks when they are detected. These can include development of best management practices, employee training regimens, and third-party certifications. Importantly, all of these innovations result in additional production costs, meaning food safety risk management would result in reduced profitability or increase prices borne on consumers. In general, when risk mitigation approaches are applied and enforced at the producer level, they reduce requirements for end-product testing (Boison and Turnipseed 2015), which can be disproportionately expensive and ineffective.

### 5.3. Regulatory level interventions

Food safety is often an important policy goal for national and regional aquaculture regulations (Naylor *et al* 2023). And, these regulations often occur at three levels: the source of the potential contaminant or pathogen, the site of production, and the point of processing, packaging, or marketing (Hammoudi *et al* 2009). Regulating sources of contaminants in aquaculture systems, especially in the context of climate change, involves a multifaceted approach. Implementing best management practices in agriculture, such as buffer zones and controlled use of fertilizers (e.g. precision agriculture), can help mitigate risk of runoff and eutrophication that can lead to HABs. Additionally, regular water quality testing for pathogens, heavy metals, and pollutants should be conducted to detect and address contamination early (Sapkota *et al* 2008). Enhancing biosecurity measures within aquaculture facilities—such as improved filtration systems and disease-resistant stock—can also reduce the risk of disease and pathogen introduction. This comprehensive approach helps safeguard the health of aquaculture systems and the safety of seafood products.

Marine spatial planning can significantly enhance the management of aquaculture systems by strategically allocating marine space to balance ecological, economic, and social objectives (Petrosillo *et al* 2023). By integrating climate change projections into marine spatial planning, planners can identify and mitigate areas at higher risk of contamination, such as those prone to HABs or pollution runoff. Such planning can help delineate zones for aquaculture that avoid regions with known contamination sources or high disease risk, thus minimizing exposure to harmful substances.

To effectively minimize contaminants and harmful exposures in aquaculture products, stringent regulatory frameworks must be established and enforced. This includes implementing comprehensive monitoring programs that routinely assess the presence of environmental contaminants, such as heavy metals, POPs, and pathogenic microorganisms, in both aquaculture systems and final products (Eguiraun *et al* 2015). Regular sampling and analysis of water, feed, and seafood should be mandated to ensure compliance with established safety standards. We propose that governments lead these monitoring programs. This top-down approach will standardize monitoring practice and reporting along with a reduction in the cost burden on producers and consumers. Additionally, aquaculture practices should be regulated to prevent contamination at the source. This involves enforcing stringent biosecurity measures to reduce the risk of disease and pathogen introduction, including the use of certified disease-free stock and maintaining controlled water quality conditions (Subasinghe *et al* 2023). Feed sources should be rigorously vetted for contaminants, and alternative feed ingredients should undergo thorough risk assessments before approval for use (Eguiraun *et al* 2015). Furthermore, the establishment of traceability systems is essential, allowing for the tracking of aquaculture products from farm to table, which facilitates prompt responses to contamination incidents and ensures accountability (El Sheikha and Xu 2017).

Incorporating Hazard Analysis and Critical Control Points (HACCP) improvements into the regulation of aquaculture products is essential for enhancing food safety (Reilly and Kaferstein 1997). HACCP systems should be regularly updated to address emerging risks and contaminants specific to aquaculture. This involves conducting thorough hazard analyses to identify and evaluate potential contamination sources, such as pathogens, chemical residues, and environmental pollutants. Critical control points in the production process should be rigorously monitored and controlled, with enhanced procedures for detecting and mitigating contamination at each stage, from feed formulation to harvest and processing. These improvements collectively strengthen the ability to prevent, detect, and respond to potential hazards, thereby ensuring the safety and quality of aquaculture products.

To minimize the risk of exposure to contaminants from aquaculture products, consumers should adopt several proactive measures. Firstly, they should stay informed about seafood sourcing and opt for products from reputable suppliers and brands that adhere to stringent safety standards and certifications. This includes checking for third-party certifications, such as those from the Marine Stewardship Council or the Aquaculture Stewardship Council, which indicate adherence to environmental and safety practices. Consumers should also practice safe food handling and preparation techniques, including thoroughly cooking seafood to recommended temperatures to kill potential pathogens and avoiding cross-contamination.

### 5.4. Research interventions

Innovative and practical research initiatives are a critical component in aquaculture’s adaptation to climate change. As local conditions are impacted by climate change, it can be difficult for farmers to adjust practices to meet shifting demands. Research aimed at identifying existing species that thrive in various aquatic environments will allow farmers to optimally select species suited to their specific environments. Additionally, novel climate-resilient species may play a pivotal role under future conditions, and efforts should focus on developing such species. Genomic approaches have already begun to identify disease-resistant loci and to establish foundations of selective breeding programs targeting more resilient species (Nguyen 2024). Similar innovations that incorporate adaptation to climate stressors will further strengthen aquaculture systems.

Research that seeks to enhance real-time hazard monitoring strategies should also be a priority. Developments in HAB monitoring will allow for longer-term and more informed decision-making by affected aquaculture operations (Stoner *et al* 2024). Additionally, recent advancements have brought real-time, low cost water quality monitoring to aquaculture farms (Nuangpirom *et al* 2025). More effective, affordable, and accessible tools will improve decision making across sectors. These research directions will be vital in addressing the complex relationship between climate change and aquaculture food safety.

## Supplementary Material

Supplementary Information S1

Supplementary Information S2

Supplementary material for this article is available online

## Figures and Tables

**Table 1.** Commodity group representation across all studies.

Commodity group	Total occurrences	Unique standardized species
Clam/Cockle	242	80
Seabass/Sea Bream	157	39
Mussel	125	15
Cyprinids (Carp/Minnows)	103	26
Oyster	99	22
Salmon	94	12
Catfish	60	26
Scallop	58	18
Shrimp/Prawn	57	5
Mullet	49	15
Whelk/Abalone/Other Gastropods	48	25
Tilapia	46	10
Crab	43	12
Lobster/Crayfish	28	10
Perch/Sunfish	21	11
Seaweed/Algae	21	19
Sea cucumber/Urchin	16	10
Snakehead	13	6
Croaker/Drum	10	4
Jack/Scad	9	3
Cod	8	1
Flatfish	8	3
Trout	8	2
Pomfret	7	3
Snapper	7	3
Eel	6	3
Other or not otherwise grouped aquatic foods	52	34

**Table 2.** Interactions of biological and chemical contaminants with climate change factors and aquaculture species affected.

Food safety risk (contaminant)	Interaction with climate change factor	Aquaculture species affected
**Aquatic biotoxins (e.g. HABs, PSPs)**	**Temperature**—affects the growth rate of certain species (Glibert *et al* 2014, Gobler *et al* 2017, Griffith and Gobler 2016). Increasing temperature affects dissolved oxygen content, leading to hypoxia (Vaquer-Sunyer and Duarte 2011). **Hypoxia**—Hypoxia is also affected by eutrophication (Roman *et al* 2019, Wallace *et al* 2014). **Extreme weather events**—(run-off from adverse weather events)—increases nutrients (Bonilla *et al* 2023) in water column for HABs proliferation (Anderson 2012, Granéli and Turner 2006, O’Neil *et al* 2012). Under hypoxic conditions induced by HABs, causes increased microbial respiration, which leads to acidification (Hallegraeff and Bolch 2016). **Acidification**—due to increased CO_2_ production by microbial activity. It can also be enhanced by eutrophication (Byrne and Przeslawski 2013, Cai *et al* 2011, Tatters *et al* 2013, Wallace *et al* 2014).	Snail mussel *(Hyropsis cumingii)* (Wu *et al* 2017) Eastern oyster (*Crassostrea virginica*) (Talmage and Gobler 2012) Pacific oyster (*Crassostrea gigas*) (Jones *et al* 1995, Talmage and Gobler 2012) Manila clams/short-neck clams (*Ruditapes philippinarum*) (Basti *et al* 2011) Northern quahog (*Mercenaria mercenaria*) (Griffith *et al* 2019) Bay scallop (*Argopecten irradians*) (Griffith *et al* 2019) Surf-clam (*Paphies donacina*) (Marsden *et al* 2016) Green-lipped mussel (*Perna canaliculus*) (Marsden *et al* 2016) Seacage salmon (*Oncorhynchus tshawytscha*) (MacKenzie *et al* 2011) Salmon (unspecified/assorted) (Samdal and Edvardsen 2020) Juvenile seabass (*Dicentrarchus labrax*) (Delegrange *et al* 2015)
**Bacteria**	**Temperature**—affects growth and proliferation of pathogenic bacteria **Salinity**—affects proliferation dynamics of pathogenic bacteria. **Acidification**—affects *vibrio spp*. hosts. Lowering pH may also affect cutaneous bacteria more than enteric species (Sylvain *et al* 2016).	Finfish: Large yellow croaker (*Pseudosciaena crocea*) (Liu *et al* 2016) Gilthead sea bream (*Sparus aurata*) (Kahla-Nakbi *et al* n.d.) Sea bass (*Dicentrarchus labrax*) (Aydin *et al* 2023) Seabass (*Lates calcarifer*) (Rosado *et al* 2021) Tilapia (*Oreochromis niloticus*) (Haenen *et al* 2023) Shellfish: Aquaculture reared phyllosoma of spiny lobster (*Panulirus ornatus,* Goulden *et al* 2012) Cultured phyllosoma of shovel-nosed lobster (*Thenus unimaculatus)* (Rehman *et al* 2022) *Penaeus monodon* (Angthong *et al* 2020), *Penaeus vannamei* (Bauer *et al* 2021), Swimming crab (*Portunus trituberculatus*) (Zhang *et al* 2014) Pacific oyster *(Crassostrea gigas)* (Gradoville *et al* 2018; Lopez-Joven *et al* 2018) *Mytilus edulis* (Ben Cheikh *et al* 2017, 2016) *Mytilus galloprovincialis* (Balbi *et al* 2019; Domeneghetti *et al* 2014)
**Parasites and viruses**	**Temperature**—affects the host’s immune response to viral infection (Groves *et al* 2023) **Salinity**—affects the survival and virality of certain viruses. Lower salinity (Garver and Hawley n.d., Tobback *et al* 2007) favors higher virality in some viruses (remain infectious longer) while higher salinity (Graham *et al* 2007) might increase the survival of other viruses. **Extreme weather events**—increased rainfall and consequent run-off may affect the increased survival and pathogen density (Coffey *et al* 2019; Colston *et al* 2022; Tornevi *et al* 2014).	Atlantic salmon (*Salmo salar*) (Groves *et al* 2023) Rainbow trout (*Oncorhynchus mykiss*) (Emmenegger *et al* 2013) Oysters (*Crassostrea gigas*) (Conaty *et al* 2000; Potasman *et al* 2002)
**Organic contaminants (e.g. POPs, pesticides, PAHs)**	**Temperature** and **extreme weather events**—affects global distillation **Salinity**—affects organisms’ ability to regulate toxicity (Noyes *et al* 2009) **UV** and **precipitation**—affects volatilization of POPs (Noyes *et al* 2009).	Atlantic salmon (*Salmo salar*) (Lundebye *et al* 2017) Mediterranean mussel (*Mytilus galloprovincialis*) (Balbi *et al* 2017) Striped catfish (*Pangasius hypopthalmus*) (Van Leeuwen *et al* 2009) Tilapia (*Oreochromis mossambicus and Oreochromis niloticus*) (Van Leeuwen *et al* 2009) Shrimp (*Penaeus monoden, Penaeus vannamei, Litopenaeus vannamei*) (Maia *et al* 2021; Van Leeuwen *et al* 2009) Trout (*Oncorhynchus mykiss, Salmo trutta*) (Van Leeuwen *et al* 2009) Largemouth bass (*Micropterus salmoides*) (Zhang *et al* 2009) Blood clam (*Tegillarca granosa*) (Tang *et al* 2020)
**Antimicrobials (e.g. antibiotics, antifungals)**	**Precipitation** and **extreme weather events**—hydrological events and land runoff increase aquatic antibiotic loading (Gomes 2024) **Temperature**—higher temperatures exacerbate effects of antibiotics and promote antibiotic resistant genes (Zhao *et al* 2024) **Salinity**—salinity gradients change antibiotic distribution, microbial community composition, and ARG prevalence (Ohore *et al* 2022)	Nile tilapia (*Oreochromis niloticus*) (Guidi *et al* 2018) Rainbow trout (*Oncorhynchus mykiss*) (Guidi *et al* 2018) Gilthead Sea Bream (*Sparus aurata*) (Santos *et al* 2016)
**Heavy metals**	**Temperature**—increased water temperature enhances the toxicity of certain metals (Pfeifer *et al* 2005; Sokolova and Lannig 2008). **Acidification**—increased accumulation of certain heavy metals in culture species (Shi *et al* 2016). **Extreme weather events**—drought increases metal concentration in fish (Azevedo *et al* 2018), while increased precipitation coupled with run-off may increase the concentration of heavy metal contaminants in the aquatic environment (Lee *et al* 2023).	Mediterranean mussel (*Mytilus galloprovincialis*) (Shi *et al* 2016) Crucian carp (*Carassius carassius*) (Lee *et al* 2023) Tilapia (*Oreochromis niloticus*) (Simukoko *et al* 2022; Yap *et al* 2015) Catfish (*Pangasius pangasius*) (Resma *et al* 2020) Rohu carp (*Labeo rohita*) (Resma *et al* 2020) Nile tilapia (*T. nolitica*) (Resma *et al* 2020) Largemouth bass (*Micropterus salmoides*) (Lee *et al* 2023) Blood clam (*Tegillarca granosa*) (Tang *et al* 2020) *Mytilus edulis* (Shi *et al* 2016) *Meretrix meretrix* (Shi *et al* 2016)
**Microplastics**	**Temperature, acidification, and UV**—increased concentration of microplastics from increased degradation of plastic pollutants under higher UV and temperature stress (Issac and Kandasubramanian 2021).	European seabass (*Dicentrarchus labrax*) (Mazurais *et al* 2015) Oyster (*Crassostrea gigas*) (Unuofin and Igwaran 2023) Mussel (*Mytilus edulis*) (Nalbone *et al* 2021; Unuofin and Igwaran 2023) Shrimp (*Metapenaeus dobsoni*) (Daniel *et al* 2021) Indian white shrimp (*Fenneropenaeus indicus*) (Daniel *et al* 2021) Crab (*Portunus pelagicus*) (Daniel *et al* 2021) Whiteleg shrimp (*Litopenaeus vannamei*) (Curren *et al* 2020) Argentine red shrimp (*Pleoticus muelleri*) (Curren *et al* 2020)

## Data Availability

We are including a list of all publicly accessible articles that have been used in this review along with our data analysis script for readers to replicate our research process. The data that supports the findings of this study are openly available in the supplementary files of this article. Supplementary information (S1) available at: https://doi.org/10.1088/2976-601X/ae854f/data1. Supplementary data (S2) available at: https://doi.org/10.1088/2976-601X/ae854f/data2. Data analysis script available at: https://github.com/wfairfield/Climate-mediated-food-safety-hazards-of-aquaculture.
