# Supplementary material for: Climate-mediated food safety hazards of aquaculture
Source: Environ Res Food Syst. Author manuscript; Available in PMC 2026 Aug 9. (PMC13449243; doi:10.1088/2976-601x/ae854f)
Supplement: Supplementary file 1 [file NIHMS2198455-supplement-Supplementary_Information_S1.docx]

**Supplementary Information – S1 – Search and Analysis Terms**

**Search Terms**

Parsimonious selection of key Title and Descriptors were rationalized by the research team to identify the corpus of relevant scientific literature. This included:

**TITLE**: ("aquaculture" OR "Seafood") and **DESCRIPTORS**: ("food safety" OR "contamination" OR "food contamination" OR "drug residue" OR “aquatic foods" OR "climate change" OR "seafood contamination" OR “food quality" OR “food hygiene" OR “drug residues" OR “bacteria" OR “ciguatera" OR “virus" OR “human health" OR “pesticide" or "biotoxin" OR “contaminant*" OR “pathogen*" OR “zoono*" OR “parasite*" OR “nanopartic*" OR “plastic*" OR “harmful algal" OR “poisoning*" OR “cyanobacteria*" OR “pesticide*" OR “mercury" OR “PFAS" OR “perfluoroalkyl" OR “endocrine disrupt*" OR “residue*" OR “trematod*" OR “nematod*" OR “cestod*")

**Analysis Terms**

## Supplementary Table 1. Wild-caught-only exclusion tags

Tags used to identify and exclude primarily wild-caught aquatic food groups or non-aquaculture residual taxa. Records matching these tags were excluded from the aquaculture-focused species and commodity-group analyses.

| **Group** | **Tags used for matching** |
| --- | --- |
| Squid | squid; loligo; illex; todarodes; dosidicus; ommastrephes; uroteuthis; nototodarus; calamari |
| Octopus/Cuttlefish | octopus; cuttlefish; sepia; sepiella; sepioteuthis; acanthosepion |
| Tuna/Bonito | tuna; thunnus; skipjack; katsuwonus; bonito; kawakawa; euthynnus |
| Mackerel | mackerel; scomber; scomberomorus; rastrelliger; trachurus; horse mackerel |
| Small Pelagic Fish (Sardine/Anchovy/Herring/Smelt) | sardine; anchovy; herring; sprat; sardina; sardinella; engraulis; clupea; hilsa; shad; tenualosa; pilchard; ilisha; capelin; clupeonella; thryssa; opisthopterus; smelt |
| Shark/Ray/Skate | shark; ray; skate; catshark; dogfish; scyliorhinus; squalus; mustelus; raja; stingray; maskray; whipray; alopias; hammerhead |
| Swordfish/Billfish | swordfish; marlin; sailfish; xiphias; sail fish; tetrapturus |
| Whitefish excluding cod | hake; merluccius; pollock; pollachius; theragra; haddock; melanogrammus; whiting; merlangius; micromesistius; ling; grenadier; coalfish; phycis; mollera; coryphaenoides; trisopterus |
| Angler/Monkfish | angler; anglerfish; monkfish; goosefish |
| Overly ambiguous taxa | fish (no species specified); shellfish (no species specified); tempura seafood (no species specified) |
| Primarily wild-caught species not captured in commodity groups | beltfish; hairtail; barracuda; whale; cutlassfish; frostfish; scabbardfish; ribbon fish; seal; narwhal; rockfish; parrotfish; alfonsin; atlantic stargazer; bichique; bombay duck; escolar; flyingfish; goatfish; sebastes; lizardfish; grunt; grunter; gurnard; needlefish; john dory; luvar; ocean jacket; oilfish; orange roughy; scorpionfish; snoek; tilefish; weever; flathead; mahi-mahi; siniperca kneri; bigeye; trumpeters fish; marlinspike auger; common periwinkle; common barbel; shaw; banded leporinus; bluering angelfish; bubblefin wrasse; indian mackerel; pacific yellowtail emperor; redcoat; thornfish; wedgespot damsel; jellyfish; false trevally; fourfinger threadfin; moontail bullseye; silver silago; brown meagre; pagel; rock goby; sand steenbras; bogue; toothed sparus; wreckfish; freshwater snail; bleak; frog triton; tuba false fusus; rabbitfish; namorado sandperch; indian spiny turbot; streaked spinefoot; common snook; banded dye-murex; changeable nassa; grooved razor shell; leerfish; mediterranean moray; painted comber; rayed trough shell; salema porgy; serrano; smooth callista; surmullet; tuberculate cockle; coral hind; melon seed; pacific saury; saury; topmouth culter; moonfish; randall's threadfin bream; conch; caitipa mojarra; thick lucine; emperor fish; bluefish; crimson jobfish; malabar trevally; minor razor shell; pod razor shell; black scraper; dark smoothhound puffer; korean sandlance; japanese sandfish; periwinkle; ponyfish; silverbiddy; patagonian toothfish; goose barnacle; pagre; silver sides; spiny top shell; nile perch; blackbelly rosefish; flexuous picarel; greater forkbeard; cisco; decapodiform cephalopod; seerfish; kurkufan; sooly; pacific ladyfish; pacific fat sleeper; kingklip; pike; horned turban; barred barb; sevenfinger threadfin; lakerda; shoemaker spinefoot fish; asp; false kelpfish; pacific sand lance; ide; podust; rudd; perch (no species specified); comber |

## Supplementary Table 2. Commodity-group allocation tags

Tags used to allocate retained aquatic food records into commodity groups for descriptive reporting.

| **Group** | **Tags used for matching** |
| --- | --- |
| Shrimp/Prawn | shrimp; prawn; penaeus; litopenaeus; fenneropenaeus; metapenaeus; macrobrachium |
| Mussel | mussel; mytilus; perna; modiolus |
| Clam/Cockle | clam; cockle; ruditapes; solenidae; venerupis; mercenaria; donax; anadara; meretrix; cerastoderma; ark shell; venus; tegillarca; cyclina; mactra; ensis; solen; lucina; callista; panopea; polymesoda; pinna; scapharca |
| Oyster | oyster; crassostrea; magallana; ostrea; pinctada |
| Scallop | scallop; pecten; argopecten; patinopecten; chlamys |
| Crab | crab; portunus; callinectes; scylla; chionoecetes; erimacrus; eocheir |
| Lobster/Crayfish | lobster; crayfish; homarus; panulirus; nephrops; procambarus; cherax; astacus; crawfish |
| Whelk/Abalone/Other Gastropods | whelk; abalone; snail; conch; gastropod; haliotis; rapana; buccinum; littorina; turbo; nerita; babylonia; murex; babylon shell; nassarius; earshell; bufonaria; terebra; bolinus; periwinkle; spotted babylon; hemifusus; tympanotonos |
| Sea cucumber/Urchin | sea cucumber; sea urchin; holothuria; apostichopus; strongylocentrotus; paracentrotus; echinoderm |
| Seaweed/Algae | seaweed; kelp; algae; alga; porphyra; saccharina; laminaria; undaria; lettuce; gracilaria |
| Salmon | salmon; salmo; oncorhynchus |
| Trout | trout |
| Cod | cod; gadus |
| Pomfret | pomfret; pampus; brama; parastromateus; trachinotus; butterfish; psenopsis |
| Cyprinids (Carp/Minnows) | carp; cyprinus; hypophthalmichthys; ctenopharyngodon; crucian; carassius; labeo; rohu; catla; mrigal; leuciscus; spinibarbus; acrossocheilus; alburnus; barbus; gobio; leptobarbus; chondrostoma; rutilus; scardinius |
| Tilapia | tilapia; oreochromis; sarotherodon |
| Catfish | catfish; clarias; pangasius; pangasianodon; ictalurus; silurus; synodontis; pseudobagrus; bagrus; keli |
| Flatfish | sole; flounder; halibut; turbot; plaice; flatfish; solea; paralichthys; hippoglossus; psetta; scophthalmus; limanda; cynoglossus; pleuronectes; megrim; paraplagusia; tonguefish |
| Seabass/Sea Bream | seabass; sea bass; bream; seabream; sea bream; dicentrarchus; sparus; pagrus; pagellus; acanthopagrus; lates; barramundi; porgy; steenbras; cephalopholis; argyrops; rhabdosargus |
| Mullet | mullet; mugil; mullus; surmullet |
| Croaker/Drum | croaker; drum; corvina; larimichthys; pseudosciaena; otolithes; sciaenops; argyrosomus; nibea; isopisthus; sciaena; scianids; macrodon |
| Grouper | grouper; epinephelus; mycteroperca |
| Snapper | snapper; lutjanus |
| Jack/Scad | jack; amberjack; scad; seriola; megalaspis; caranx; lichia; trevally |
| Perch/Sunfish | perch; bluegill; lepomis; siniperca; mandarin fish; walleye; sander; zingel |
| Eel | eel; anguilla; conger; muraena; muraenesox; kingklip |
| Snakehead | snakehead; channa |
| Flathead | flathead; platycephalus |
| Other or Not Otherwise Grouped Aquatic Foods | Assigned when no commodity-group tag is detected. |

## Supplementary Table 3. Species-level wild-caught exclusion tags within retained commodity groups

Tags used to identify specific primarily wild-caught taxa within otherwise retained commodity groups. These taxa were excluded from the aquaculture-focused species and commodity-group analyses.

| **Group** | **Tags used for matching** |
| --- | --- |
| Shrimp/Prawn | atlantic northern shrimp; atlantic seabob; bay shrimp; chinese mud shrimp; deep water shrimp; deep-water rose shrimp; fiddler shrimp; golden shrimp; grass prawn; japanese mantis shrimp; jawla paste shrimp; jinga shrimp; king prawn; mantis shrimp; ocean shrimp; oriental shrimp; prawn; rainbow shrimp; red shrimp; ridgeback shrimp; salad shrimp; spear shrimp; torpedo shrimp; whiskered velvet shrimp; white prawn; white shrimp; yellow shrimp |
| Crab | brown crab; common moon crab; european spider crab; japanese stone crab; light-blue soldier crab; mangrove swimming crab; orchid crab; pacific stone crab; red king crab; samoan crab; sand crab; sand swimming crab; snow crab; spider crab; spotted crab |
| Cod | hound needlefish; pacific cod; poor cod |
| Pomfret | chinese pomfret; golden pomfret; pomfret; silver pomfret |
| Flatfish | adriatic sole; bengal tonguesole; bloch's tonguesole; common dab; doublelined tonguesole; european plaice; foureyed sole; greenland halibut; halibut; joyner's tongue-sole; largescale tonguesole; lemon sole; megrim; tongue sole; oriental sole; plaice; red tongue sole; ridged-eye flounder; shotted halibut; sole; southern flounder; thickback sole; wedge sole |
| Croaker/Drum | banded croaker; belanger's croaker; big-snout croaker; bighead croaker; bronze croaker; croaker; distinct croaker; lesser tigertooth croaker; pama croaker; panna croaker; reeve's croaker; sin croaker; sinna croaker; soldier croaker; southern kingcroaker; tigertooth croaker; white mouth croaker; yellow croaker |
| Grouper | banded grouper; grouper; snowy grouper; spinycheek grouper; starspotted grouper; white grouper |
| Snapper | emperor red snapper; humpback red snapper; red snapper; russell's snapper; southern red snapper; yellow snapper |
| Jack/Scad | bermuda jack; bigeye scad; bigeye trevally; bluefin trevally; indian scad; japanese scad; ocean jacket; oxeye scad; redtail scad; shortfin scad; striped jack; torpedo scad; yellowstripe scad; yellowtail scad |
| Eel | conger eel; conger pike; european conger; rosy eel goby; snake-eel; pink cusk-eel |
